# Environmental Enrichment Extends Photoreceptor Survival and Visual Function in a Mouse Model of Retinitis Pigmentosa

**DOI:** 10.1371/journal.pone.0050726

**Published:** 2012-11-28

**Authors:** Ilaria Barone, Elena Novelli, Ilaria Piano, Claudia Gargini, Enrica Strettoi

**Affiliations:** 1 Neuroscience Institute, Italian National Research Council (CNR), Pisa, Italy; 2 G.B. Bietti Foundation for Ophthalmology, Rome, Italy; 3 Department of Pharmacy, University of Pisa, Pisa, Italy; University of Florida, United States of America

## Abstract

Slow, progressive rod degeneration followed by cone death leading to blindness is the pathological signature of all forms of human retinitis pigmentosa (RP). Therapeutic schemes based on intraocular delivery of neuroprotective agents prolong the lifetime of photoreceptors and have reached the stage of clinical trial. The success of these approaches depends upon optimization of chronic supply and appropriate combination of factors. Environmental enrichment (EE), a novel neuroprotective strategy based on enhanced motor, sensory and social stimulation, has already been shown to exert beneficial effects in animal models of various disorders of the CNS, including Alzheimer and Huntington disease. Here we report the results of prolonged exposure of rd10 mice, a mutant strain undergoing progressive photoreceptor degeneration mimicking human RP, to such an enriched environment from birth. By means of microscopy of retinal tissue, electrophysiological recordings, visual behaviour assessment and molecular analysis, we show that EE considerably preserves retinal morphology and physiology as well as visual perception over time in rd10 mutant mice. We find that protective effects of EE are accompanied by increased expression of retinal mRNAs for CNTF and mTOR, both factors known as instrumental to photoreceptor survival. Compared to other rescue approaches used in similar animal models, EE is highly effective, minimally invasive and results into a long-lasting retinal protection. These results open novel perspectives of research pointing to environmental strategies as useful tools to extend photoreceptor survival.

## Introduction

Retinitis pigmentosa (RP) comprises a group of inherited retinal disorders with an incidence in humans of approximately 1/3500 [1], [2]. The genetics of RP is complex: 180 mutations [3] in 43 known genes have been associated with the autosomal dominant, recessive and X-linked forms of RP [4], [5] [https://sph.uth.tmc.edu/retnet/sum-dis.htm]. In all cases rods undergo progressive degeneration, causing night vision loss. Subsequently, cones, responsible for high-acuity daytime vision also die, thus leading to blindness.

Although no cure for RP is presently available, different therapeutic approaches have been translated into clinical application. A promising treatment is based upon neuroprotection, which aims to delay the intrinsically slow degenerative process of RP by interfering with the death of rods, given the finding that increased rod survival extends cone viability as well [6]. Because of the importance that cones have for everyday life, and considering that as little as 10% of the total cone number is sufficient for independent living in humans [1], developing treatments finalized to extend the lifespan of these cells are particularly important. Clinical trials are currently testing the efficacy of intraocular devices releasing ciliary neurotrophic factor, CNTF [7]–[9]. Still, the optimal combinations of neurotrophic factors to be delivered are to be defined [10], [11] and the best dosages must be found to maintain appropriate regulation of neurotrophin levels avoiding adverse effects [12].

A novel neuroprotective approach shown to achieve remarkable effects in animal models of various CNS affections is environmental enrichment (EE), defined as the exposure to an environment combining social and inanimate stimuli, increasing motor, sensory and social activities [13]. EE was associated with significant reductions in disease progression and symptoms in rodents with experimental ischemia [14], Huntington chorea [15] and Alzheimer disease [16]–[20]. The visual system is especially sensitive to EE, which accelerated retinal and visual cortical development [21]–[23], increased visual acuity in mice [24] and completely reverted experimental amblyopia in rats [25]. EE stimulated the generalized production and release of neurotrophic factors, e.g. NGF, BDNF and NT-4, throughout the CNS [26]–[28]. BDNF has been indicated as one of the mediators of the effects of EE on the visual cortex [23], [26] and the retina [29]. Consequently, we investigated whether EE translates into a neuroprotective effect on inherited photoreceptor degeneration, possibly by raising retinal levels of endogenous protective factors.

We studied the effects of EE in rd10 mice, in which a mutation of the beta subunit of the rod-specific phosphodiesterase gene leads to well-characterized rod-cone degeneration. Here, rd10 mice were born and raised in EE; afterwards, their retinal structure, visual function and performance and expression of molecular markers were compared to those of age-matched rd10 mice raised in standard laboratory conditions (ST). We found that rd10 exposed to EE had improved photoreceptor viability, more preserved retinal morphology and better visual function over time.

## Methods

### Ethics Statement

All procedures were performed according to the guidelines of the Italian Ministry of Health for care and maintenance of laboratory animals (law 116/92), and in compliance with the European Communities Council Directive n. 86/609/EEC. Animal experimentation at the CNR Neuroscience Institute was approved by the Italian Ministry of Health (authorization # 129/2000−A). The experimental protocol described in this study was specifically authorized by the Italian Ministry of Health (decree #185/2009-B, released on 11/04/2009). Retinal tissue was always obtained from animals deeply anesthetized with intraperitoneal injections of avertine, also use for ERG recordings. Animals were killed with anaesthetic overdose after eye removal. All efforts were made to minimize suffering. Mice were rd10 mutants (B6.CXB1-Pde6brd10/J, on a C57Bl6J background) [30] and wild-type (C57BL/6J) (wt), both from Jackson. The presence of the homozygous Pde6b mutation was assessed periodically by PCR on DNA extracted from tail tissue as explained in [31]. A total number of 180 rd10 mice (90 kept in ST and 90 in EE conditions) were used for this study. A number of 16 additional wild type mice of the C57Bl6J strain were used for control experiments.

**Figure 1:**
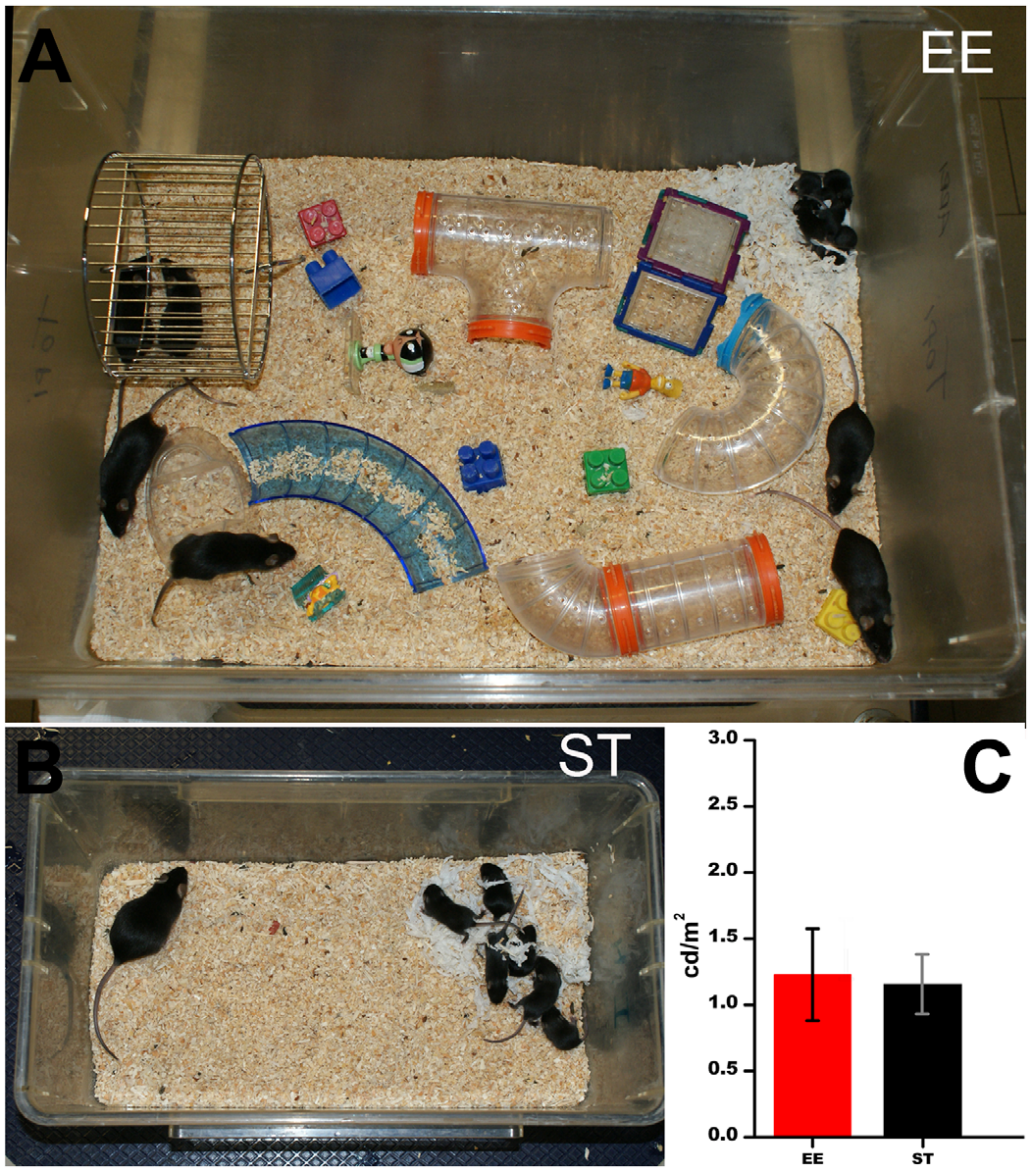
Enriched (EE) and standard conditions (ST). (A) EE cages have a complex design, with multiple objects and access to free, voluntary movement. Groups of mice comprise 5 adults and one litter. Control mice are kept in the same room but in standard (ST) laboratory cages (B). Both EE and ST cages have stainless steel grid lids. As shown in the diagram in C, ambient light levels measured with a radiometer placed in different positions inside various cages do not differ in EE and ST conditions (n = 7 readings per cage, 4 cages for each experimental condition; data are shown as means and SEM). Tunnels are transparent and objects small so the animals cannot escape light by hiding behind them.

**Environmental Enrichment (EE)** cages consisted of large Plexiglas boxes (60×38×20 cm^3^) containing a metal running wheel 12–18 cm in diameter, 2 transparent plastic tunnels (15–22 cm long and 6 cm wide), nesting material (small pieces of thin tissue paper), and 6–8 small hard-plastic objects (about 3×7 cm) (see Figure 1). Running wheels were repositioned once a week and tunnels and objects were replaced with the same frequency [22]. To raise rd10 mice in conditions of EE, a fertile female was placed for 7 days in an enrichment cage with a male and 4–5 older parous females (helpers). With this procedure, the whole pregnancy occurred in EE. The male was removed on the day of birth of the new litter. The rd10 progeny (usually 6–8 pups) remained in the same cage for the whole period of testing (up to 210 days), while on day 30 the mother and helpers were removed. Control animals were rd10 mice born and raised in standard laboratory conditions (ST). These animals were kept in the same room, on the same shells and same diet (food and water *ad libitum*) of EE mice, but in standard Plexiglas cages (36×20×13 cm^3^) without objects except for some tissue paper at the time of nest building. These cages hosted only the pregnant female and, later, her litter (see Figure 1). Additional control animals were wt mice having the same genetic background (C57Bl6J) as the rd10 strain; these animals were raised in ST only. Since all the experiments were performed on animals of the same background strain, the differences observed cannot be attributed to strain effects.

### Illumination Conditions

These were identical for both EE and ST mice, and consisted of a 12-hr artificial light-dark cycle, with average illumination levels of 1.2 cd/m^2^ provided by two white neon lamps placed on the room ceiling. Light measurements were obtained inserting in the cages a CS 100 Minolta Chroma radiometer pointing outside the 4 cage walls and to the top of them (Figure 1C). The tunnels used for enrichment cages were transparent and did not cut light intensity significantly with respect to average values. Running wheels were made of thin metal wire, were fixed to the cage bottom and could not shield the light. Because the light source was on the room ceiling, perpendicular to the cages, the shadow projected by the objects was extremely small.

### Retinal Morphology and Photoreceptor Counts

Whole eyes for different experimental protocols were obtained from mice kept in EE and ST on 45, 60 and 75 days of age. A total of 15 mice per condition (5 for each age group) were used for immunocytochemistry (ICCH) on vertical retinal sections. For this purpose, whole eyes were immersions-fixed in 4% paraformaldheyde in 0.1 M phosphate buffer, rinsed, sucrose-infiltrated, frozen at −20°C and serially sectioned at 14 µm on a cryostat. Immunostaining on vertical sections and confocal microscopy (Leica TCS-NT and TCS-LP confocal microscopes) were performed as explained in [32]. Rod and cone morphologies were studied by using antibodies against rhodopsin (Sigma O4886, 1∶250), cGMP-gated light-sensitive channel (a gift from J. Beavo, University of Washington, Seattle, USA; 1∶1000), cone red/green (L/M) opsin (Millipore AB5404), cone blue (S) opsin (Millipore AB5407), and recoverin (Millipore AB5585), all used at 1∶500 dilution. The morphologies of rod bipolar cells, horizontal cells, cone bipolar cells and synaptic markers in the outer plexiform layer were studied using antibodies respectively against PKCα (Sigma, P4334 and P5704; 1∶1000), calbindin-D-28K (Swant CB-38a, 1∶2000), postsynaptic density protein 95 (PSD95; Abcam, ab13552, 1∶500), bassoon (Abcam, ab76065; 1∶1000) and mGluR6 (a gift from S. Nakanishi, Kyoto University Faculty of Medicine, Japan, used at 1∶2000). Secondary antibodies were anti-rabbit and anti-mouse immunoglobulins conjugated with Alexa Fluor 488, 568 (from Invitrogen) or with Rhodamine Red-X (from Jackson Immuno Research). They were used at a dilution of 1∶1000. The overall survival of photoreceptors was assessed on vertical sections by counting the number of rows in the outer nuclear layer after fluorescent nuclear staining (using 2 µM ethidium homodimer 1, or YOYO-1, both from Invitrogen) and confocal microscopy. Equatorial sections (i.e. including the optic nerve head) of eyes from ST and EE mice were sampled systematically acquiring images of the outer nuclear layer (ONL) at central, midperipheral and peripheral locations. Number of rows in the ONL, and ONL thickness were measured with the aid of Metamorph® version 5.0r1 (Molecular Devices).

**Cone survival** was determined at 45, 60 and 75 days of age using an original quantitative method that takes into account local anisotropies in cell distribution, common in retinal degenerative pathology, and samples the whole retinal surface along the two main meridians. Cones were counted in retinal whole mount preparations (n = 15 per experimental condition, 5 retinas from different animals for each age group) stained with antibodies against S and M/L opsins (from Millipore). Incubation in primary antibodies (diluted 1∶200) lasted 3 days; after extensive rinsing in PBS, retinas were incubated for additional 2 days in Alexa 568 conjugated secondary antibodies diluted 1∶1000. After rinsing in PBS, retinas were mounted on glass slides photoreceptor-side up, covered with Vectashield (from Vector) and coverslipped. Subsequently, retinas were imaged with a Zeiss fluorescence microscope interfaced with an Axiocam CCD camera. A 10× objective was used. Since cone outer segments lie in a thin focal plane, images of stained retinal whole mounts provide high-resolution topographic maps which, in the case of rd10 mice, show bright cone-rich areas alternating with dark degenerating zones. Digital montages of images of the whole retinal surface were obtained using the Photomerge routine of Adobe Photoshop CS4 and thresholded with Metamorph to better isolate cone clusters. Using differences in brightness displayed on the images as color gradients, cone isodensity curves were traced digitally. Cone densities within each curve were calculated separately by counting cones on high-resolution confocal images obtained at each retinal location. For that, 2 images of the focal plane encompassing cone outer segments, each covering 125×125 square micrometers, were collected at the confocal microscope at 16 retinal locations, spaced along the dorso-ventral and naso temporal meridians and located within all the isodensity regions identified on low power micrographs of each retinal sample. Cones were counted on these images with an object recognition routine of Metamorph. The total number of cones was finally obtained by multiplying local densities by the corresponding areas of isodensity regions and summing up the results. Considering that the sampled area encompasses approximately 3.8% of the retinal surface, the method of counting used here can be considered particularly accurate.

**Figure 2:**
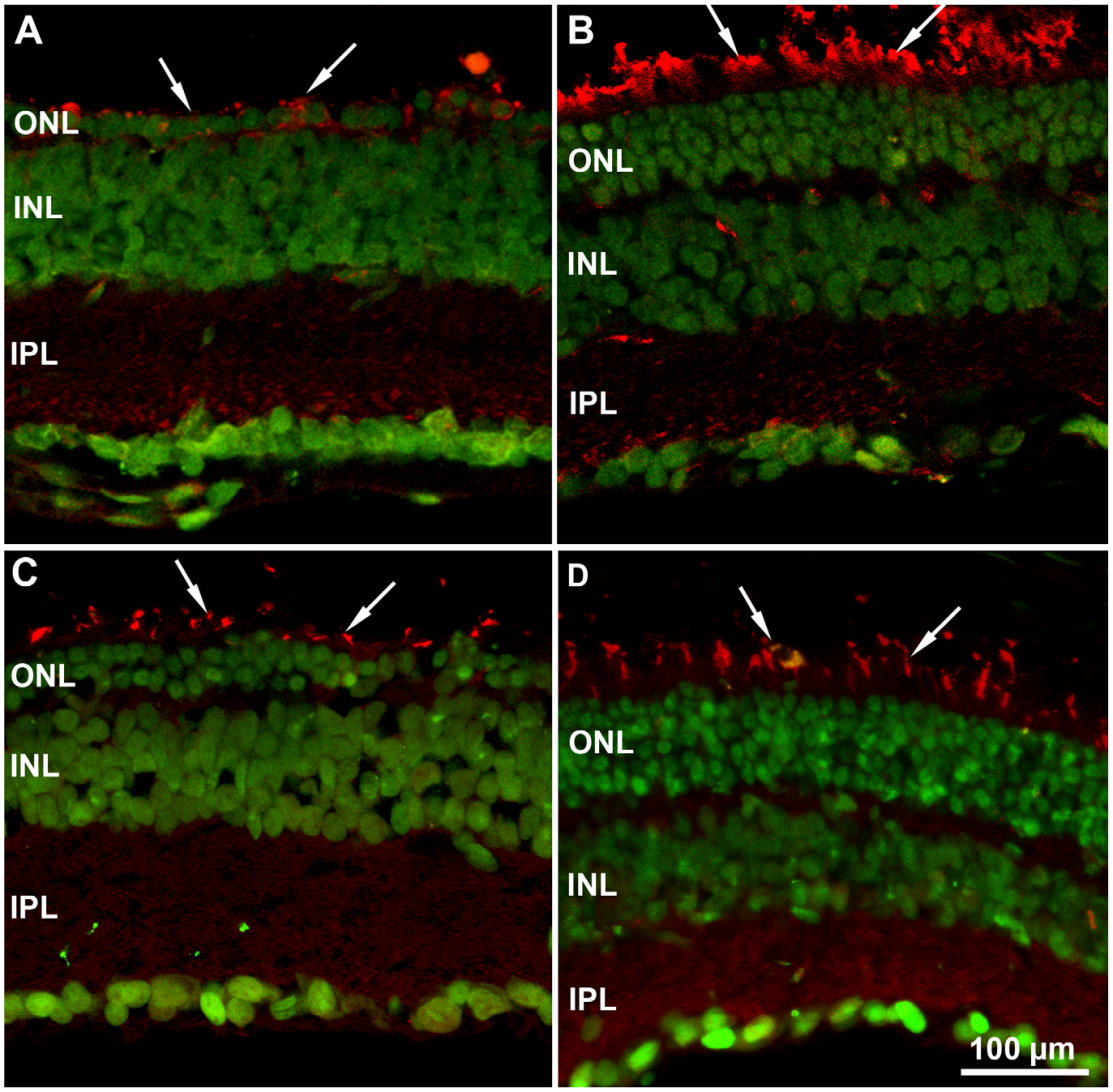
Photoreceptor survival and morphology in mice raised in ST and EE. (A to D) Vertical retinal sections from 45 (A, B) and 60-day-old (C, D) rd10 mice raised in standard conditions (ST) or in environmental enrichment (EE). Rhodopsin staining (red) and nuclear counterstaining (green). Extensive rod degeneration and pronounced outer segment loss in ST (A). EE (B) is associated with a greater number of nuclear rows and preservation of rod outer segments (arrows). Compared here are two samples (A and B) of central retina, where photoreceptor degeneration is faster. (C and D) Cone-opsin staining (red) and nuclear counterstaining (green). Note preservation of cone outer segments (arrows) in EE (D) compared to ST (C). C and D are taken from peripheral retina, where photoreceptor degeneration is slower. Here and in the following pictures: ONL, INL and IPL: outer nuclear, inner nuclear and inner plexiform layer, respectively.

### Western Blot

Retinal homogenates were obtained from 3 EE and 3 ST mice aged P60, using both retinas of each animal for each preparation. Loading on gel was done in duplicate per sample. Protein extracts (60 µg) were separated by electrophoresis and blotted on PVDF membranes that were subsequently incubated with the following antibodies: anti-rhodopsin (the same used for immunostaining), anti-S and M/L opsins (OPN1SW, H-40 and OPN1MW/LW, H-55; from Santa Cruz Biotechnology, used at 1∶2000 dilution), anti-β-actin (Sigma, A2228; 1∶2000) and anti α-tubulin (abcam, ab4074; 1∶5000), which served as an internal standards for protein quantification. Filters were then incubated in secondary antibodies, which were immunoglobulins conjugated with HRP (Bio-Rad), and developed by ECL (Bio-Rad). Film quantification was done after optical scanning measuring band density with Quantity Basic Biorad system. Band readings were normalized to corresponding values of β-actin or α-tubulin used as loading controls. Comparisons were made between mean values.

**Figure 3:**
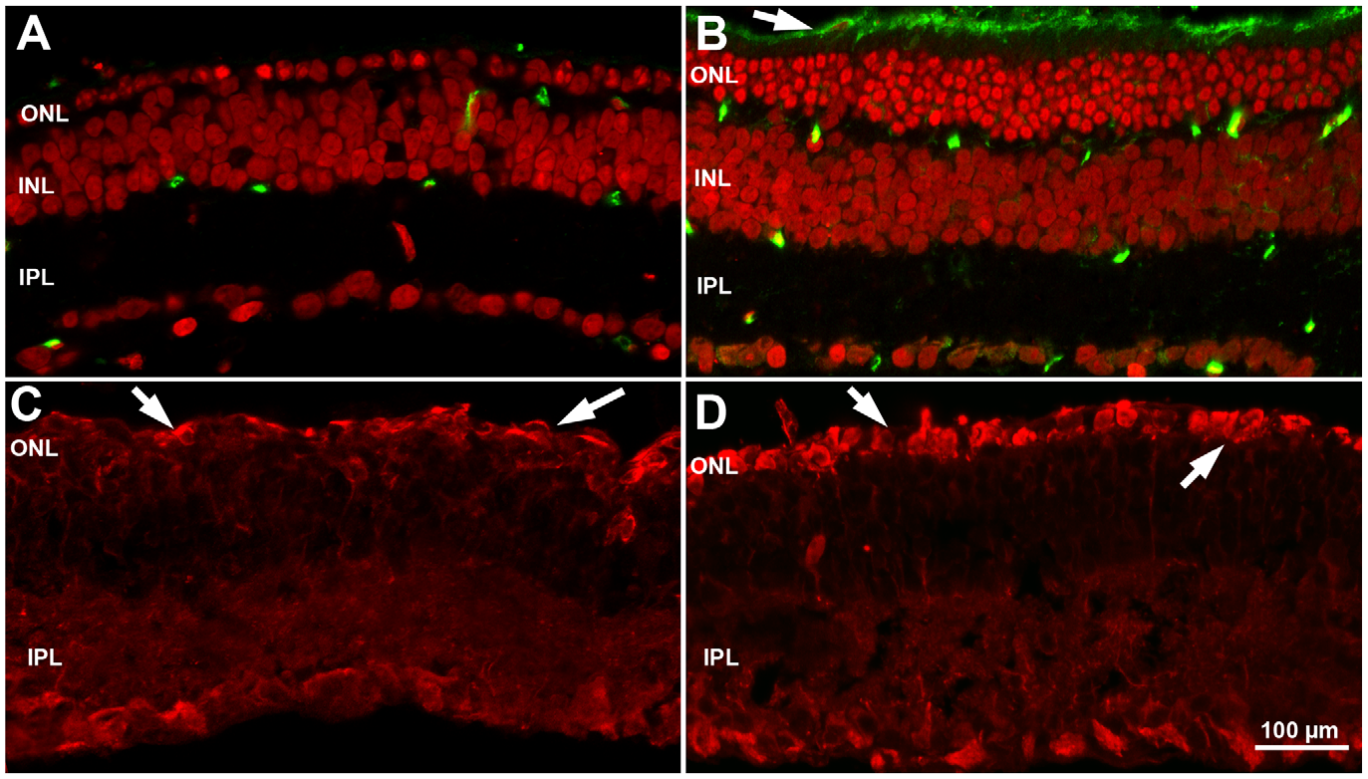
Photoreceptor specific markers in EE and ST. (A, B) cGMP-gated light-sensitive channel staining of rods (green) and nuclear counterstaining (red). Good rod outer segment preservation in EE (arrow in B) compared to ST (A). Age is P45. (C and D). Central retina. Recoverin staining (red). Higher number of photoreceptors (arrows) in EE (D). Age is P60.

**Flash Electroretinograms** (ERGs) were recorded as described in [32] from dark-adapted anesthetized mice at P45, P60 and P75 (n = 24 mice, 8 for each age group, for each experimental condition). An electronic flash unit generated a light stimulus of 492 nm whose energy decayed with a τ of 1.7 ms. Full-field stimulation was achieved using a Ganzfeld sphere. Mice were subjected to 8 different flash intensities (from 0.09 to 377.23 cd*s/m^2^), each repeated 5 times, with an inter stimulus interval that ranged from 20 s for dim light to 1 min for the brightest flashes. Since the responses to the brightest flashes include mixed rod and cone components, isolated cone components (photopic ERGs) were obtained by superposing all test flashes on a background of saturating intensity for rods (30 cd/m^2^). ERG signals were recorded using coiled gold corneal electrodes. Responses were differentially amplified, bandpass filtered at 0.3–500 Hz, digitized at 0.25- to 0.5-ms intervals and stored on disc for processing. Five ERG traces at each flash luminance were averaged before measurements of cone b-wave amplitudes. The amplitude of the b wave was taken as the difference between baseline and the peak value.

**Figure 4:**
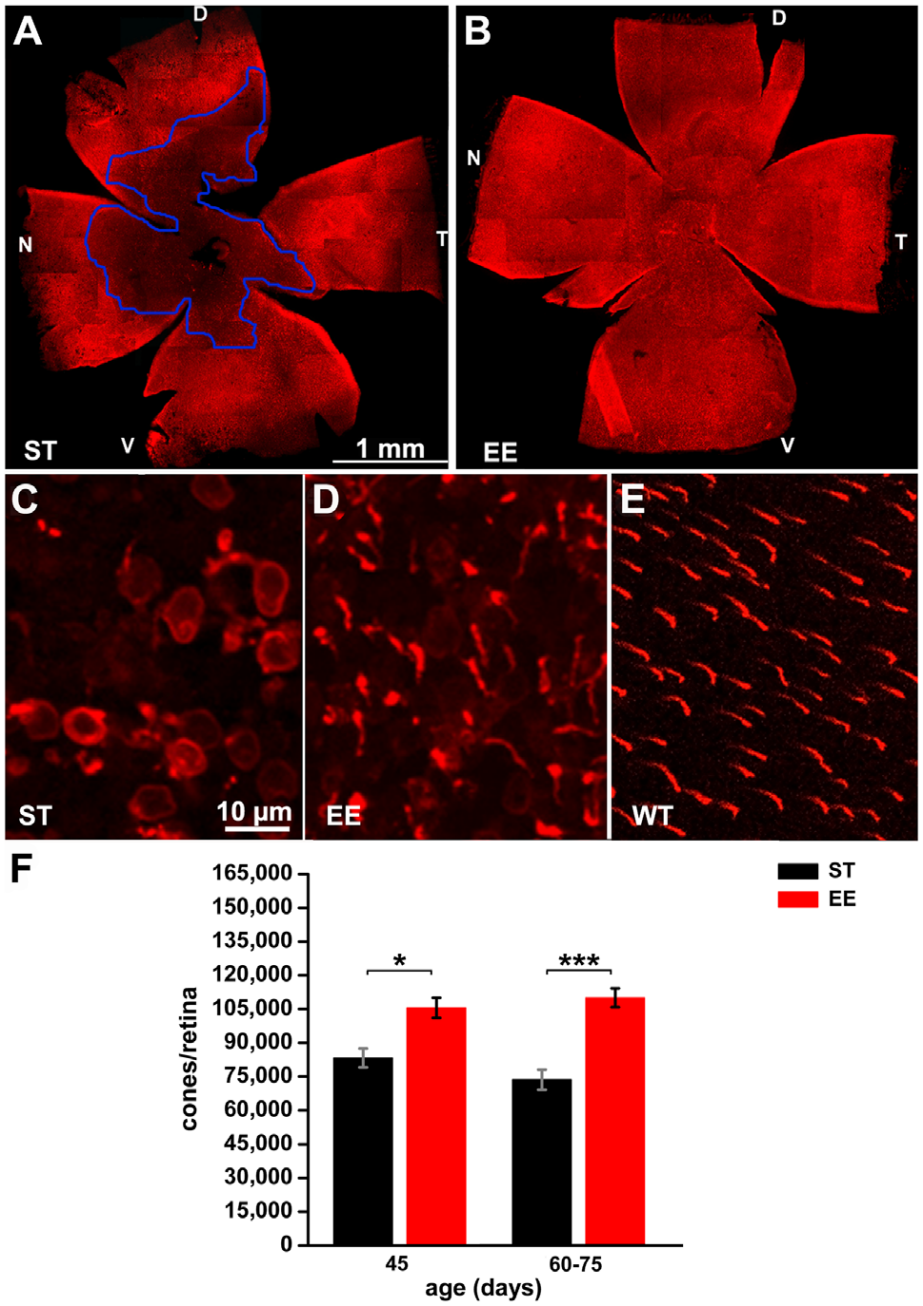
Morphology and survival of cones in mice raised in ST and EE. Retinal whole mounts from 60-day-old rd10 mice raised in ST (A) and EE (B), stained with antibodies against S and M/L cone opsins (red). An area devoid of cones is evident in the central part of the retina shown in A (blue line). Cones are still preserved in B. Density values ranged from 3,000 and 50,000 cones/mm^2^ in this ST retina versus 4,000 to 85,000 cones/mm^2^ in the EE preparation. D, V, N, T: dorsal, ventral, nasal and temporal poles of the retina. (C to E) High-magnification confocal images of retinas stained for cone opsins from rd10 mice raised in ST (C) or in EE (D) and from wildtype mice (E). Residual cones in ST have lost outer segments (C), still considerably long in EE (D). (F) Counts of cones in retinas from rd10 mice raised in ST (black bars) and EE (red bars) at 45 (n = 4 per condition) and 60–75 days of age (minimum n = 5 per condition). The number of cones decays markedly in retinas from mice in ST from P45 to P75, whereas it remains constant in mice raised in EE. Values are mean of means and SEM. Student’s *t* test. *p = 0.011; ***p<0.001.

### Visual Behaviour

Visual acuity was assessed at 45, 60, 90, 120, 150 and 210 days of age under photopic conditions using a water maze developed by Prusky [33]. In this task mice learn to swim in a pool 15 cm deep, filled with tepid (22°C), opaque water, toward a submerged platform whose position is indicated by an above-water computer screen showing a black and white grating. A blank (grey) computer screen with the same main luminance of the grating serves as decoy. Stimuli were computer-generated square-wave black and white gratings having a fixed luminance of 39.95 cd/m^2^ with spatial frequencies ranging between 0.087 and 0.550 cycles/degree. After the mice learned to associate the platform’s location with the grating at low frequency, the frequency of the grating was increased, thereby increasing the number of parallel bars in the animal’s angular field of view (increased cycles/degree). The highest spatial frequency perceived (permitting discovery of the escape platform with a success rate above 70% of the trials) defines the upper limit of visual acuity. This was measured in ST and EE mice tested progressively from P45 to P210 (n = 12 mice for each condition).

**Figure 5:**
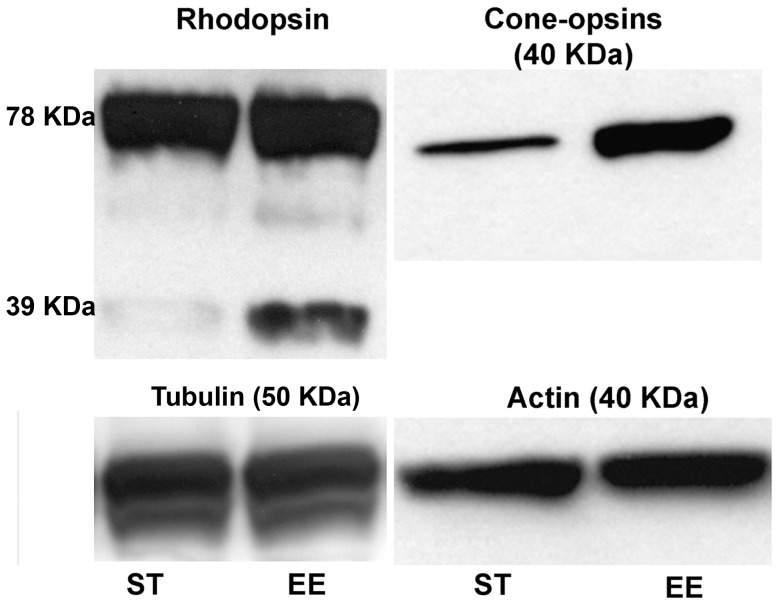
Examples of Western blots of rhodopsin and cone-opsins in retinal homogenates from rd10 mice raised in ST and EE. Age is P60.

The visual water task was used on the same mice to measure the threshold for contrast detection using gratings with progressively decreasing contrast (from 90% to 1%) but fixed frequency (0.117 cycles/degree) and luminance (39.95 cd/m^2^). The lowest contrast that permitted the mice to still locate the platform with a success rate above 70% of the trials was recorded as a marker of their contrast sensitivity. This was done for rd10 EE and ST mice, as well as for wt animals, at P45, P60 and P90. Comparisons for both visual acuity and contrast sensitivity were made between mean values using a Student T test. Since the same animals were tested at different time, two way ANOVA analysis followed by Bonferroni test were carried on to evaluate the effects of both age and treatment upon both visual parameters.

### qRT-PCR

To quantify mRNA levels of BDNF, NGF, IGF1, CNTF and mTOR, total RNA was isolated from frozen retinas from ST and EE mice aged P13 (n = 4), P45 (n = 8) and P60 (n = 12) using QIAzol Lysis Reagent (Qiagen). Genomic DNA was removed and RNA was reverse transcribed using the QuantiTect Reverse Trascription Kit (Quiagen). For each sample, 0.5 µg RNA were used for retrotranscription. Quantitative real-time PCR was performed on an Applied Biosystems StepOne thermal cycler using TaqMan qPCR Master Mix 2X (Applied Biosystems).

**Figure 6:**
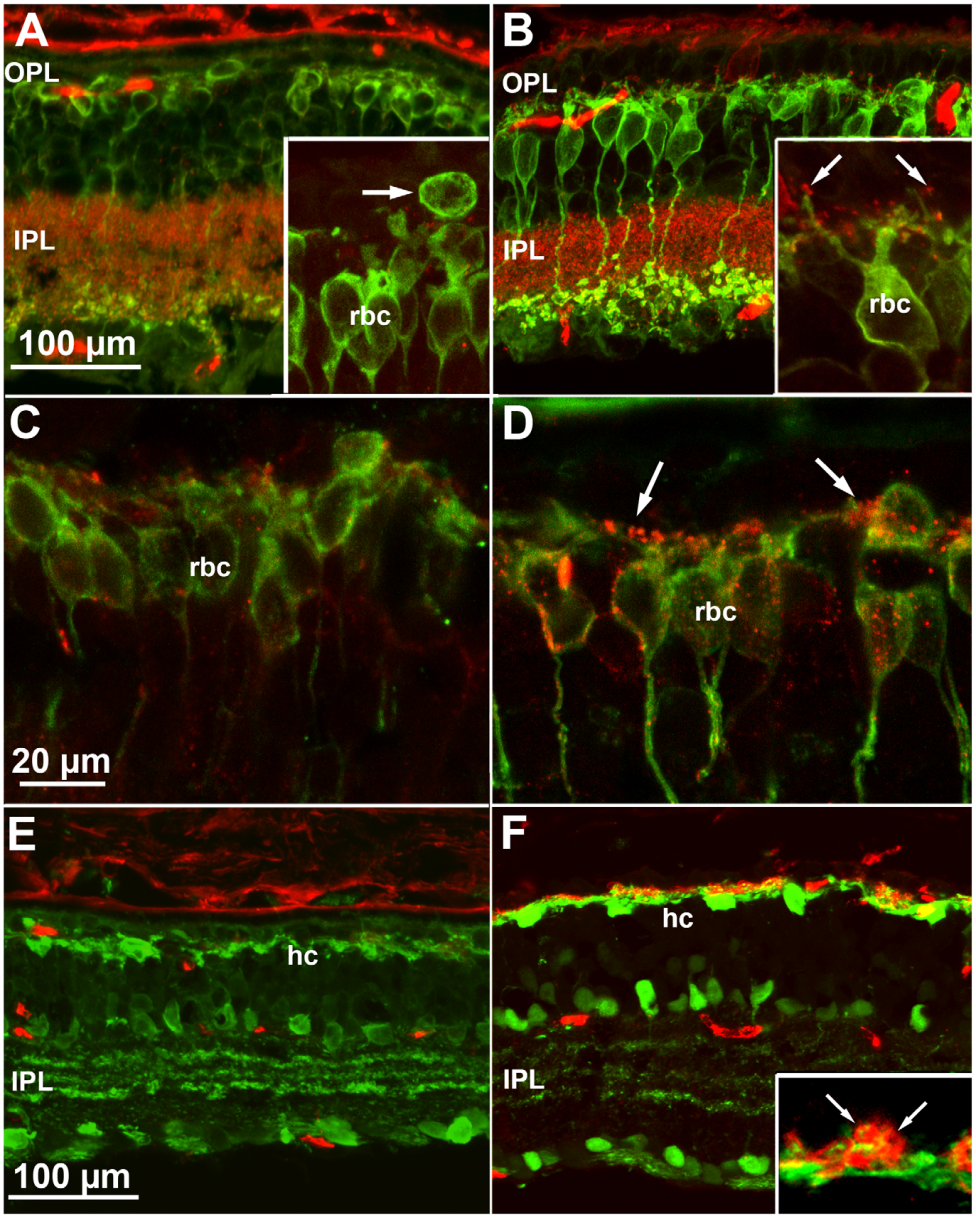
Morphology of inner retinal cells in mice raised in ST and EE. (A and B) Vertical retinal sections stained with antibodies against PKCα (green), labelling rod bipolar cells (rbc), and with antibodies against bassoon (red) that, in the outer plexiform layer (OPL), label synaptic contacts established by photoreceptors. Age is P60. In ST there is pronounced dendritic retraction of rbc (A) while in EE these are better preserved (B). Also in EE, dendrites are decorated by bassoon-positive puncta (B, arrow in insert) that are virtually absent in ST (A, insert; the arrow points to the displaced cell body of a rbc). (C and D) mGluR6 (red) and PKC staining (green) show that rod bipolar cells, in EE retinas (D) maintain longer dendrites and a complement of mGluR6 (arrows), which are rare in ST counterparts (C). Age is P60. (E and F) Calbindin-D-28 staining of horizontal cells (hc, green). In ST (C), horizontal cell processes in the outer retina are scant and poorly ramified. In contrast, in EE (D), the still branched dendrites of these neurons are surrounded by photoreceptor synaptic endings, labelled by anti-PSD95 antibodies (red), which highlight the round shape of their terminals (inset). Insets are shown at twice the magnification of the main panels.

The probes used for quantitative real-time PCR were all TaqMan® Gene Expression Assays (20×) as reported below: GADPH: Mm99999915_g1*; NGF: Mn00443039_m1; BDNF: Mm01334047_m1; mTOR: Mm00444968_m1; CNTF: Mm00446373_m1. Glyceraldehyde-3-phosphate-dehydrogenase (GADPH) was used as a reference gene to calculate relative transcript levels. Samples from each animal (two retinas combined) were assayed in triplicate for each gene tested. Relative quantification values were derived using the delta-delta Ct method. Results obtained in EE were normalized to those in ST.

**Figure 7:**
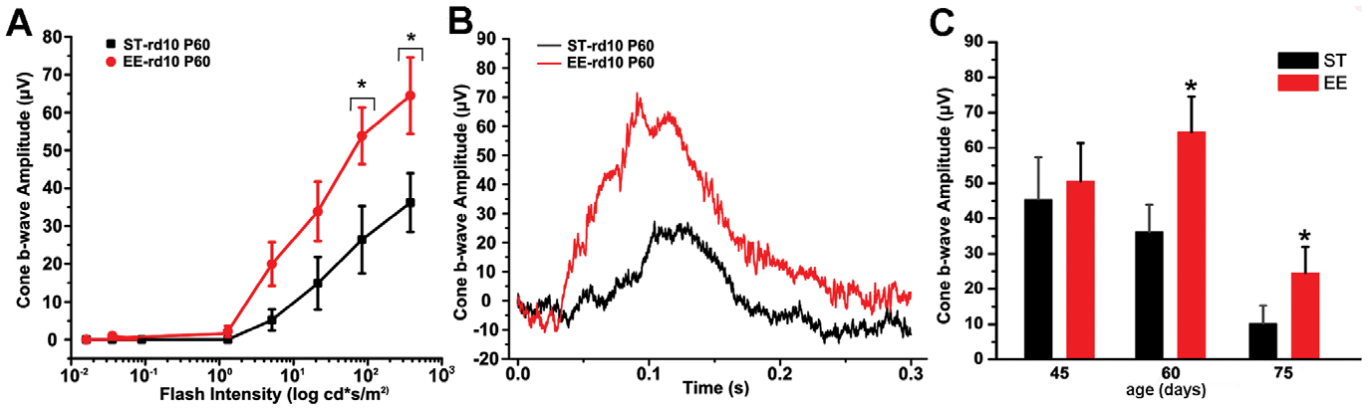
Retinal physiology in mice raised in ST and EE. (A) Photopic ERG responses recorded at P60 and plotted as a function of the flash intensity. At high intensities, amplitudes are significantly greater in mice raised in EE (*p<0.05). Bars indicate SEM. (B) Representative photopic ERG traces from ST and EE rd10 mice aged 60 days, at high luminance. (C) b-wave amplitudes of photopic ERG responses at high luminance (377,23 cd*s/m^2^) were similar between EE (red bars) and ST groups (black bars) at 45 days of age but were significantly more preserved in EE at 60 and 75 days, when cone degeneration is widespread in the rd10 strain (Student’s *t* test, *p = 0.02).

## Results

In rd10 mice, rod loss starts around post-natal day 18 (P18), peaks at P24 and is almost complete by P40 [32]. Cone death proceeds more slowly, and the cone-mediated electroretinogram (ERG) is extinguished gradually by P70–P75. As previously described, mice kept in EE open their eyes 2 days before their counterparts in ST [21]. This was found to be true also for rd10, enriched mice, which were observed to undergo eye opening (defined as the initial break in the membrane sealing the lids of both eyes) at postnatal day 12 (P12), rather than at P14, as ST control pups. Behaviorally, enriched mice were observed to explore objects and tunnels and to access the running wheel frequently (Figure 1), especially during the night hours.

**Figure 8:**
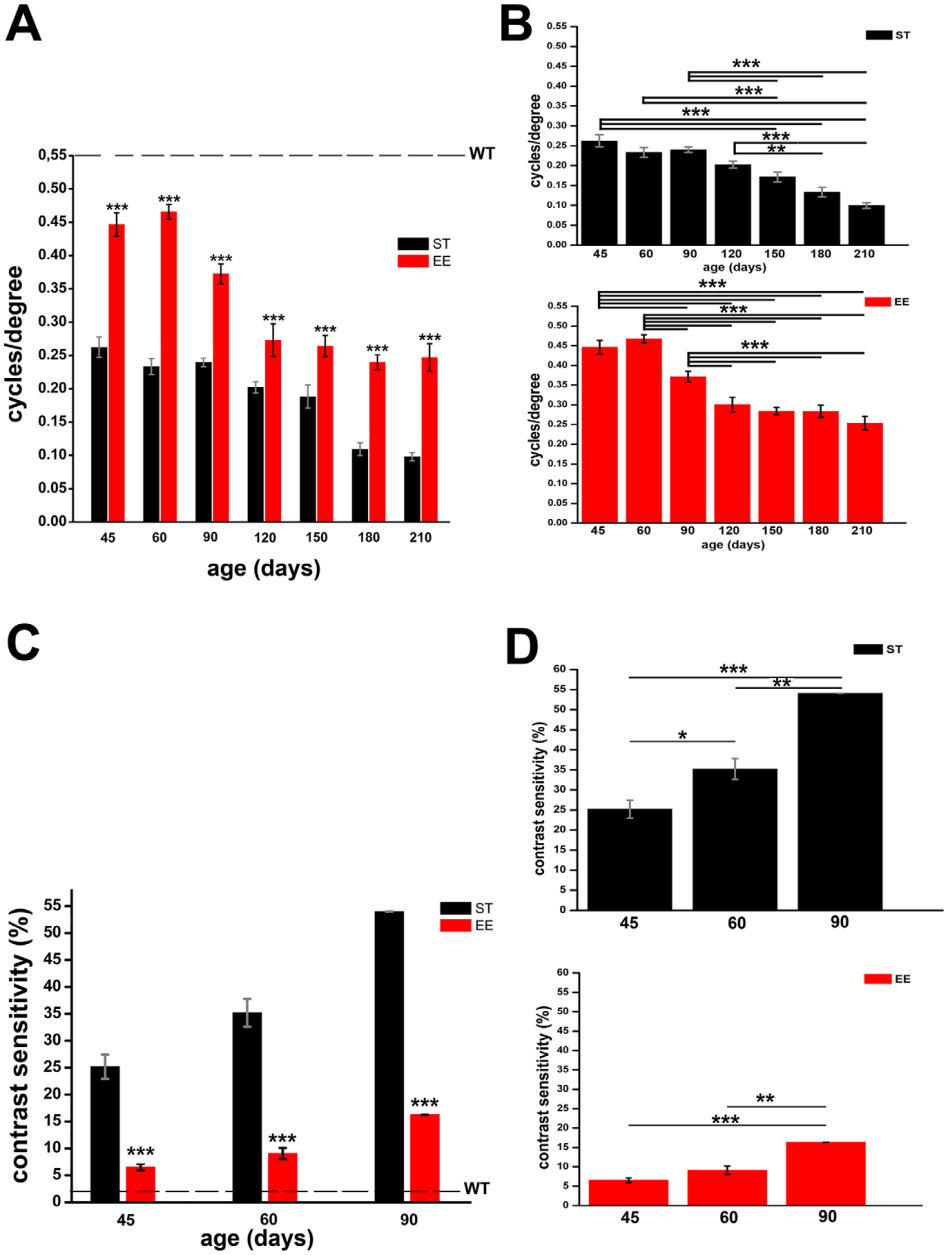
Visual behaviour in ST and EE mice. (A) Visual acuity of rd10 mice is significantly higher in EE than in ST at all ages tested. Two-way ANOVA test with Bonferroni post-hoc analysis, ***p<0.001. Each column shows the mean (and SEM) response for a group of 10 animals tested with a Prusky water maze. The valued shown for the wild type strain (dotted line) is the one reported in [21]. (B) Visual acuity of EE (upper panel) and ST mice (bottom panel) as a function of time. Two way Anova test and Bonferroni correction show that visual acuity decays more rapidly in ST than in EE mice. (C) At P45, contrast sensitivity, assessed as the minimum contrast detected, is already poor in rd10 mice raised in ST, and becomes worse with age. rd10 mice raised in EE have better contrast sensitivity than ST counterparts, although showing an age-related decline. Two-way ANOVA test with Bonferroni post-hoc analysis, ***p<0.001. The value shown for the wild type strain (dotted line) is the one reported in [37]. (D) Again, two-way Anova matched and Bonferroni correction highlight the age dependent decline in contrast sensitivity. ***p<0.001; **p<0.01; *p<0.05.

### Photoreceptor Survival and Morphology

As in ST mice, and in a fashion typical for RP mouse models, photoreceptor degeneration in EE mice followed a central-to-peripheral topography and was observed first in rods and then in cones. However, in retinas of rd10 mice born and raised in EE, the number of rows of surviving photoreceptors in the outer nuclear layer was consistently higher than in rd10 mice maintained in ST at all post-natal ages tested (up to P75). Between P45 and P60, only 1–2 (+/−1, SE) rows of photoreceptor nuclei were found in the peripheral retina of ST mice, while 4–6 rows (+/−1, SE) persisted at similar eccentricity in the retina of rd10 mice in EE (Figure 2A–B). Since these comprise cells that could be stained with antibodies against rhodopsin and cone specific opsins, they included both rods and cones (Figure 2). Even in the central retina, where degeneration was faster, a continuous row of photoreceptors was observed in preparations from EE rd10 mice, while these cells appeared intermittent in the outer nuclear layer of retinas from ST controls (Figure 2, C and D; Figure 3, C and D). In EE, but not ST, rod outer segments showed positive immunostaining for rhodopsin (Figure 2, A and B) and the cGMP-gated light-sensitive channel (Figure 3, A and B). Recoverin staining was also present (Figure 3, C and D), indicating that surviving photoreceptors still expressed phototransduction proteins. Although degeneration was still evident in the retinas of EE rd10 mice, this was much more limited than that in rd10 ST counterparts.

**Figure 9:**
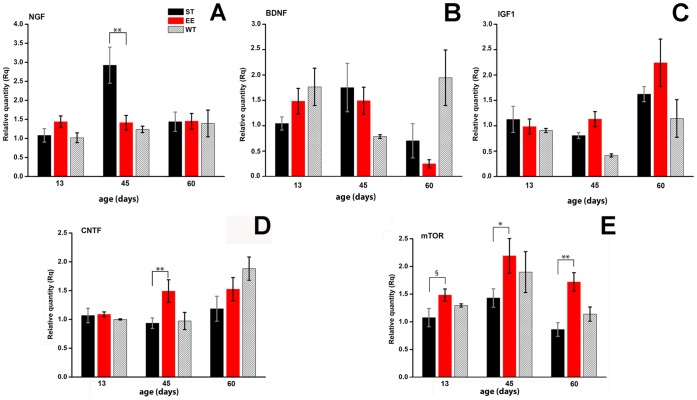
qRT-PCR on EE and ST retinal samples. A–C Relative amounts of mRNA for NGF, BDNF and IGF-1 detected by qRT-PCR in retinal extracts from rd10 mice raised in ST (black bars) and EE (red bars) at 3 different ages. Gray bars refer to age matched wild type mice also kept in ST. For all the assays, Rq values (referred to GADPH mRNA) in EE were normalized to those in ST. One way Anova with Bonferroni post-hoc analysis. Columns show average values +/− SEM. It is to note that the decrease of NGF observed in EE rd10 mice at 45 days of age (p = 0.006) is only with respect to ST controls but not in comparison to age-matched wild type mice. D. Relative amounts of mRNA for CNTF and mTOR detected by qRT-PCR in retinal extracts from rd10 mice raised in ST (black bars) and EE (red bars) at 3 different ages. Extracts were from groups of 8–10 retinas for each age. Columns show average values +/− SEM. Statistic differences calculated by one-way Anova with Bonferroni post-hoc analysis are given in the text.

**Cone survival** was assessed on retinal whole mounts stained with antibodies against blue and red/green cone opsins (Figure 4). At P60, in ST control rd10 mice (Figure 4A), an area devoid of cones was clearly evident in the central retina, according to the centre-to-periphery pattern of degeneration typical of rodents. Instead, in EE rd10 mice, degeneration was much milder (Figure 4B). At higher magnification, the cones in ST appeared aberrant and ablated of outer segments, with opsin immunoreactivity diffused to the cell membrane (Figure 4C). In EE (Figure 4D), surviving cones had better preserved morphology and longer outer segments, although shorter than in wt mice (Figure 4E). Using a newly developed procedure that quantifies cone density across the whole retinal surface, we found over 100,000 cones persisting in retinas of EE in the time range P45–P75 (Figure 4F), corresponding to 56% of the cone population of a normal mouse retina [34], while about 80,000 aberrant cones (44% of that in wildtype mice) survived in retinas from age-matched mice in ST. It is to note that the method we used for counting takes into account both cones retaining an outer segments and cones devoid of this portion; however, we noticed that many of the cones from ST retinas had lost their outer segment.

Maintained survival and preservation of rods and cones in EE rd10 mice was confirmed by western blot analysis. Quantification of retinal levels of the same photoreceptor-specific proteins documented by immunostaining (rhodopsin, and S and M/L cone opsins) showed that these were higher in EE than in ST (Figure 5). The average value of rhodopsin (both the monomer and the dimeric form, 39 and 78 KDa, respectively) measured on 2 sets of blots from 4 EE and 4 ST animals, was 35% higher in EE, while cone-opsins were 20% higher.

### Inner Retinal Cells in EE and ST

It is well known that at early stages following photoreceptor death, the so called phase I remodelling comprises regressive changes in second order retinal neurons. Typically, arborizations from cells postsynaptic to rods (rod bipolar and horizontal cells) retract extensively while photoreceptor endings and their synaptic machinery in the outer plexiform layers are progressively lost [35]. Indeed, in ST, regressive events were seen as pronounced dendritic retraction of rod bipolar cells and loss of immunoreactivity for bassoon, a protein labelling synaptic ribbons in photoreceptor terminals (Figure 6A). In EE, rod bipolar cell dendrites were more extensively ramified and were decorated by bassoon-positive puncta (Figure 6B). Similarly, dendrites of depolarizing bipolar cells retained immunoreactivity for mGluR6, the particular glutamate receptor which mediates synaptic transmission from photoreceptors (Figure 6, C and D). Well organized horizontal cells were visualized by calbindin staining in EE retinal preparations, as opposed to horizontal cells with atrophic dendrites in age-matched ST preparations (Figure 6, E and F). Anti-PSD95 antibodies, showing the contours of photoreceptor endings in the outer plexiform layer, demonstrated that these were also more preserved in EE than in ST (Figure 6, E and F). Thus, EE was effective in limiting the regressive remodelling of retinal neurons postsynaptic to photoreceptors that is typically associated with the death of these cells.

### Visual System Physiology in EE and ST

The improved photoreceptor viability of rd10 mice exposed to EE was confirmed by recordings of the photopic Electroretinogram (ERG). The amplitude of the b-wave as a function of light stimulus intensity in ST and EE animals is reported in Figure 7A. Records in B are examples of responses obtained at high luminance. Collected results from the two groups of animals at different ages are illustrated by the diagram in C. In EE mice, the amplitude of the b-wave at P60 and P75 was significantly higher than in age matched ST animals. These results demonstrate that the surviving cones observed in EE were functionally viable.

To determine if the cellular and ERG improvements achieved by raising rd10 mice in EE translated into improved visual perception, we used the visual water task, behavioural test, to assess spatial frequency and contrast sensitivity. In this test, the highest spatial frequency of a grating displayed on a computer monitor and perceived by the animals defines the upper limit of visual acuity. Mice raised in EE had significantly higher visual acuity at all time points, with residual vision at 210 days (Figure 8A and B). The speed with which acuity decayed in time was slower in EE compared to SD animals.

In the same animals, we also measured the contrast sensitivity, i.e. the lowest value of contrast that still permitted the mice to recognize a grating of fixed frequency (Figure 8 C and D). At P45, mice raised in ST had poor contrast sensitivity (requiring a minimum of 25% contrast to locate the platform) and this deteriorated noticeably over time (54% at P90). Instead, mice raised in EE had significantly better contrast sensitivity at all three time points (detecting gratings with only 7% contrast at P45 and 16% at P90), although here too there was a decline with age. In agreement with previously published data, wt mice of the C57Bl6J strain, also raised in ST conditions and used as additional controls, had the highest contrast sensitivity, requiring 2% contrast to perform the task [36], [37]. It is to note that EE and ST rd10 mice did not show any difference in the average number of trials necessary to learn pattern discrimination in this task. The task training phase was always performed at the age of 24 days, when cone degeneration is still limited in rd10 animals.

Altogether, these results point to the efficacy of EE in extending considerably the time window during which rd10 mice can effectively see.

### qRT-PCR on Retinal Extracts from EE and ST rd10 Mice

To determine whether the effects of EE were modulated by increased production of neurotrophins or other factors known to be increased by EE and/or to sustain survival of photoreceptors, we selectively measured retinal levels of BDNF, NGF, CNTF and mTOR mRNA by qRT-PCR. Relative levels of NGF and BDNF were similar between mice raised in ST and EE at P13, P45 and P60, with the exception of NGF, which was higher at P45 (Figure 9A and D). Instead, CNTF mRNA was similar in EE and ST at P13 and P60 but this pattern was very different at P45, when relative levels of CNTF were higher in EE compared to ST controls (p = 0.0028: one-way Anova with Bonferroni post-hoc analysis) (Figure 9D). mTOR was higher in EE samples at all the age tested (Figure 9E), with a particularly net increase at P60 (one-way Anova with Bonferroni post-hoc analysis, p = 0.001), the age at which cone death is known to be conspicuous in the rd10 mutant. Since EE has been shown to increase blood levels of insulin-growth factor 1 (IGF-1) [38], which, in turn, stimulates mTOR, we also measured IGF-1 mRNA in retinal extracts but we found no variations at all the age tested (Figure 9C).

## Discussion

In human RP as well as in its animal models, photoreceptors are destined to die and near blindness is the inevitable outcome. So far, no treatment other than replacing, when possible, the defective gene can definitely stop the progression of the disease. However, neuroprotection might slow it down. In this study, rather than developing a pharmacological approach to RP, we applied the technique of environmental enrichment (EE) in which experimental animals were born and raised under conditions that stimulated voluntary motor activity, sensory experience and social interactions. This manipulation produced remarkable therapeutic effects on the visual system by significantly extending the time during which rd10 mice, doomed to early blindness, maintained good visual functions.

All measurements carried out to test the effects of EE were consistent in showing significant improvements with respect to ST environmental conditions: morphological studies documented increased photoreceptor survival and maintenance of their connectivity; biochemistry demonstrated preservation of photoreceptor-specific proteins; ERG recordings confirmed retention of retinal capability to respond to light; visual acuity, which is set in principle by the cone density but that also reflects the overall visual system performance, was measured in a behavioural task, which confirmed preservation of cone-mediated vision; finally, contrast sensitivity, indicating the integrity of the neuronal processing of visual signals [37], demonstrated conserved visual function and visual perception abilities over time. Thus, in agreement with the results obtained for other CNS pathologies, EE also has substantial benefits on inherited retinal degeneration. Compared to other rescue strategies tested in the same animal model [39]–[42], EE is non invasive and results into long-lasting effects. It is also likely to be mutation-independent.

It is conceivable that EE acts by enhancing retinal self-defence mechanisms. In diseased retinas, several trophic factors (e.g. CNTF, FGF-2) are chronically upregulated [43], [44]. Major local sources of trophic molecules include Muller cells [45], [46], astrocytes [47], activated microglia [48], and the retinal pigment epithelium [49]. Upregulation of trophic factors is a homeostatic response probably enhanced by both the disease itself and EE, as shown by the increased levels of CNTF found here in correspondence with the active phase of cone degeneration. CNTF is the most effective of all factors tested for the ability to protect photoreceptors in a large variety of retinal diseases [9]. Intracellular signaling following binding of CNTF to its receptor can activate directly survival through the pathway based upon the Phosphatidylinositol 3-kinase (PI3K) [50]. Another candidate of the pro-survival effects produced by EE is mTOR, whose expression was enhanced in enriched conditions. mTOR stimulation through the insulin pathway has been shown to prolong sensibly the lifetime of cones reducing the shortage of glucose that leads to their starvation [51]. mTOR is a sensor of nutrient and growth factor levels and controls, among others, protein synthesis and cell growth. The increased metabolism associated to enhanced physical activity typical of EE might be the cause of increased mTOR levels in the retina. A raise in blood levels of IGF-1 caused by elevated metabolism might not translate into increased mRNA levels of this factor in the retina but signals that increase mTOR are anyway multiple. For instance, the cascade involving PI3K/AKT(PKB) triggered by CNTF may activate itself the mTOR pathway that stimulates translation [50]. Thus, both direct and combined actions of retinal CNTF and mTOR might contribute to the observed enhancement of photoreceptor survival in EE. Obviously, the beneficial effects of EE on vision, measured behaviourally, are also likely to involve extra retinal changes, some of them happening in the visual cortex.

To our knowledge, this is the first time that a direct link is established between EE and raised levels of CNTF and mTOR mRNAs in the retina or elsewhere in the CNS. No previous attempts were done to employ EE as a strategy to delay retinal degeneration. Obviously, the mediators responsible for the positive effects of EE on photoreceptor degeneration are yet to be clarified. Diffusible factors capable of crossing the retinal blood barriers might be enhanced by EE, which is known to affect both general metabolism and gene expression in local brain areas [52], eventually triggering a pro-survival response in photoreceptors. Moreover, metabolites that are actively transported through the blood-brain barrier can act as signalling molecules at retinal level. Alternatively, retinal cells, like dopaminergic amacrines, sensitive to overall ambient light could release factors that in turn support photoreceptors [53].

In future, we plan to dissect out which components of EE (motor, sensory, cognitive, or social stimulation) are mostly responsible for enhanced survival of photoreceptors, similarly to what has been done for visual cortical effects of EE [54]. Because mice are mostly active at night, it is likely that motor, rather than visual stimulation plays a crucial role in promoting the protective effects of EE. In addition, given the increased levels of maternal care associated to an enriched environment (23), the influence of maternal components upon the favourable outcome of EE on retinal degeneration should be investigated. Notwithstanding the unknown mechanism responsible for preservation of photoreceptors in enriched conditions, the non-invasive nature of EE makes this approach particularly interesting for further exploration. This manipulation of the external milieu could be considered a strategy to delay symptoms in human patients when other treatments are not available or must be postponed. Translation of EE from the laboratory to human RP individuals is not immediate, as humans are believed to live in already complex environments and to commonly experience novelty. And yet, the exposure to cognitive, social and physical stimulation is highly variable among individuals and, for a single person, changes throughout the course of a lifetime. Moreover, EE favours stress-free and challenge-free interactions in a stimulating surrounding that might enhance self-defence responses of the body to disease [21], [23]. Recently, it has been shown that visual field and visual acuity are considerably more variable in RP patients with reduced physical functioning, indicating an effect of life habits on retinal and visual performance [55]. The results reported here should encourage the exploration of EE paradigms as a tool to delay RP symptoms in view of a definitive cure. Aspects of the life style that could be recommended to human patients are regular physical exercise and social interactions, often sacrificed by limitations imposed by or associated to a blinding disease. Preservation of the visual function through an intensified use of the sensory, motor and cognitive systems could be a novel and non invasive possibility to promote neuroprotection, which could be assessed on “enriched” and control patients enrolled in a trial in which, for instance, physical exercise and cognitive stimulation are provided in a controlled fashion. This is particularly interesting considering the finding that EE is effective in delaying the degeneration of cones, the only photoreceptors upon which RP patients rely and the most important for human vision.
